# Successful Pregnancy Following Ovarian Platelet-Rich Plasma (PRP) Treatment, the DuoStim Protocol, and Preimplantation Genetic Testing for Aneuploidy in a Patient With Premature Ovarian Insufficiency: A Case Report

**DOI:** 10.7759/cureus.98505

**Published:** 2025-12-05

**Authors:** Flavia Costanzi, Ermanno Greco, Sara Fusco, Gianluca Dani, Giuseppe Grimaldi, Maria Alba Stigliano, Ilaria Listorti, Pierfrancesco Greco

**Affiliations:** 1 Centre for Reproductive Medicine, Villa Mafalda, Rome, ITA; 2 Surgical and Medical Sciences and Translational Medicine, Sant'Andrea Hospital, Sapienza University of Rome, Rome, ITA; 3 Centre for Reproductive Medicine, ICSI Roma New Life, Rome, ITA; 4 Complex Operational Unit of Immunohematology and Transfusion Medicine (UOC SIMT), Blood Component Production Centre (CPE) Local Health Authority Rome 1 (ASL Roma 1), Rome, ITA

**Keywords:** genetic testing for aneuploidy (pgt-a), intraovarian platelet-rich plasma, platelet-rich plasma (prp), poi, premature ovarian insufficiency

## Abstract

Premature ovarian insufficiency (POI) severely limits reproductive options. We report a successful live birth in a patient with POI following a combined approach of intraovarian platelet-rich plasma (PRP) treatment, double ovarian stimulation (DuoStim protocol), and preimplantation genetic testing for aneuploidy (PGT-A). PRP treatment was performed to restore ovarian function, after which DuoStim was used to maximize oocyte yield. An embryo obtained underwent PGT-A, and transfer of a euploid blastocyst resulted in an uncomplicated pregnancy and the birth of a healthy child. This case highlights the potential of personalized and multidisciplinary strategies to overcome the traditional barriers in POI by integrating regenerative medicine (PRP), advanced ovarian stimulation (DuoStim), and genetic embryo selection (PGT-A). While this innovative approach appears promising for women with diminished ovarian reserve, further studies are needed to confirm its efficacy and safety.

## Introduction

Premature ovarian insufficiency (POI) is a complicated and multifaceted disease in reproductive medicine. The condition is defined by loss of ovarian activity before the age of 40 years and is characterized by amenorrhea or irregular menstrual cycles with elevated gonadotropins and low estradiol in accordance with the European Society of Human Reproduction and Embryology (ESHRE) guidelines [1]. The diagnosis is generally made based on amenorrhea, high gonadotropins, and low anti-Müllerian hormone (AMH) levels [1]. Because POI profoundly limits spontaneous fertility and the effectiveness of conventional assisted reproductive technologies (ARTs), it represents a major clinical challenge.

Novel attempts have been made in recent years to overcome the scarce reproductive solutions available to women with POI [2]. Intraovarian infusion of platelet-rich plasma (PRP) appears to be a technique that may enable the recovery of ovarian function by promoting high concentrations of growth factors, cytokines, and chemokines in ovarian tissue [3]. These bioactive factors are thought to increase cell proliferation and tissue regeneration and favor a microenvironment for folliculogenesis organization, thereby increasing ovarian parameters [4]. This approach remains experimental but has attracted growing clinical interest.

Regarding the type of action, two principal hypotheses have been advanced. The less controversial concept is neo-oogenesis, which is believed to reflect the presence of ovarian stem cells that allow adult ovaries to produce new oocytes [5]. Various studies have demonstrated that mitotically active germ cells can be obtained from healthy adult ovarian tissue in both mice and humans [6,7]. Nevertheless, it remains unclear at this point whether spontaneous activation of stem cells occurs in the adult human ovary.

Another potential interpretation is that the PRP procedure induces and matures dormant or quiescent primordial follicles, leading to more follicles being available for ovulation [5]. The substantial biological and methodological differences between mouse and human tissues suggest that this treatment modality should be considered cautiously for application in humans [5]. Furthermore, more complex ovarian stimulation protocols, such as DuoStim (double stimulation in the same cycle), are currently applied to maximize the number of retrieved oocytes in poor responder patients [8].

Moreover, the addition of preimplantation genetic testing for aneuploidy (PGT-A) further favors embryo selection and subsequent transfer, as well as the final chance of pregnancy, especially in women with diminished ovarian reserve (DOR) [9]. Furthermore, how PRP affects embryonic euploidy rates is unclear.

Here, we report a case in which PRP combined with DuoStim and PGT-A led to euploid blastocysts and live births in a patient who had POI. This case contributes additional evidence to the emerging role of regenerative and advanced reproductive techniques in the management of POI.

## Case presentation

A couple presented to the Centre for Reproductive Medicine at Clinica Villa Mafalda, Rome, Italy, due to the onset of POI [1]. The couple reported a three-year history of secondary infertility and, upon anamnesis, denied any significant medical conditions, smoking, or other notable factors. Genetic evaluations confirmed the absence of cystic fibrosis mutations and normal karyotypes; fragile X carrier screening revealed normal results, no mutations in coagulation genes (factors V, II, or MTHFR), and no thalassemia or glucose-6-phosphate dehydrogenase (G6PD) mutations. The male partner, aged 40 years, had semen analysis showing normal parameters according to the WHO 2021 criteria [10]: a volume of 1 mL, concentration of 56 million/mL, motility with type A at 3%, type B at 30%, type C at 17%, normal morphology at 4%, and pathological forms at 96%. The terminal deoxynucleotidyl transferase-mediated dUTP nick end labeling (TUNEL) test for DNA fragmentation was 4% (negative), confirming a diagnosis of normospermia.

The female patient was 39 years old with five months of amenorrhea. The hormone profile at the start of the procedure was as follows: AMH 0.03 ng/mL, follicle-stimulating hormone (FSH) 90 mIU/mL, and luteinizing hormone (LH) 15 mIU/mL, with a confirmed POI diagnosis. Transvaginal ultrasound revealed no antral follicles on either ovary, and the endometrial thickness was 4.7 mm. The results of the general blood analyses revealed the following: thyroid-stimulating hormone (TSH) at 1.4 UI/mL, thyroid antibody negativity, and autoimmune antibody negativity.

PRP treatment

Two cycles of intraovarian PRP therapy are recommended for patients who have signed a consent for treatment [11,12]. Preoperative examinations, including routine blood tests, ECG, smear tests, and cervical-vaginal swabs, were conducted. PRP was prepared from 20 mL of peripheral blood, which was divided into two 10 mL tubes, via a RegenLab Kit (RegenLab SA, Le Mont-sur-Lausanne, Switzerland). Each tube underwent a single centrifugation at 1,500 rpm for five minutes at room temperature. From each tube containing 10 mL of centrifuged blood, 2 mL of PRP was obtained per tube. The resulting PRP was activated with 10% calcium gluconate before being injected. Intravenous sedation with 100 mg of propofol was administered. Then, 2 mL of PRP per ovary was gradually injected into the ovarian subcortical and stromal areas via a transvaginal ultrasound-guided approach through a 17-gauge, 30 cm-long Cook single-lumen needle (Cook Medical, Bloomington, IN, USA). The patient received antibiotics and tolerated the procedure without experiencing any pain or adverse effects.

**Figure 1:**
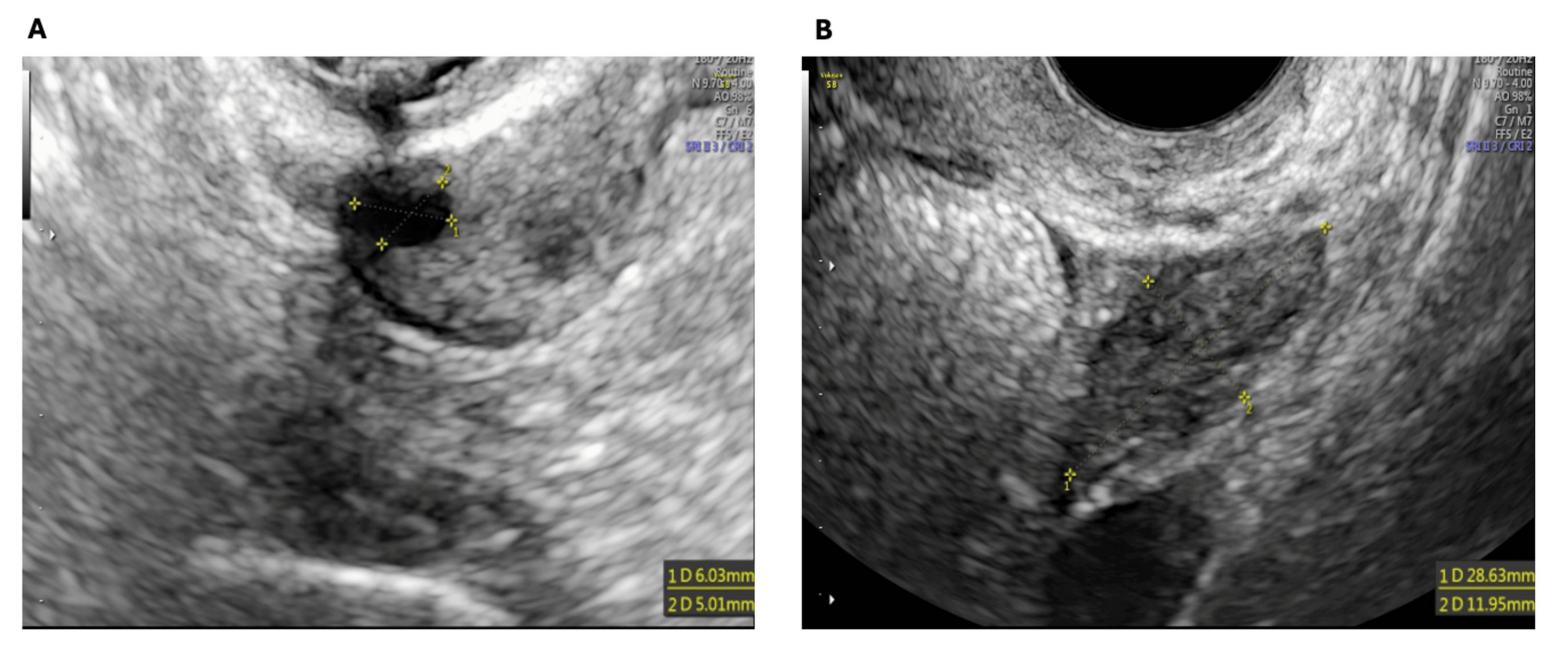
Ovarian ultrasound findings one month after the first PRP treatment. (A) Transvaginal ultrasound of the right ovary showing a single antral follicle measuring 6 mm in diameter at one month post-initial PRP administration. (B) Transvaginal ultrasound of the left ovary demonstrating absence of antral follicles at the same time point. PRP: platelet-rich plasma

One month after the first PRP treatment, an ultrasound examination revealed a 6 mm follicle in the right ovary and no antral follicles on the left (Figure 1).

**Figure 2:**
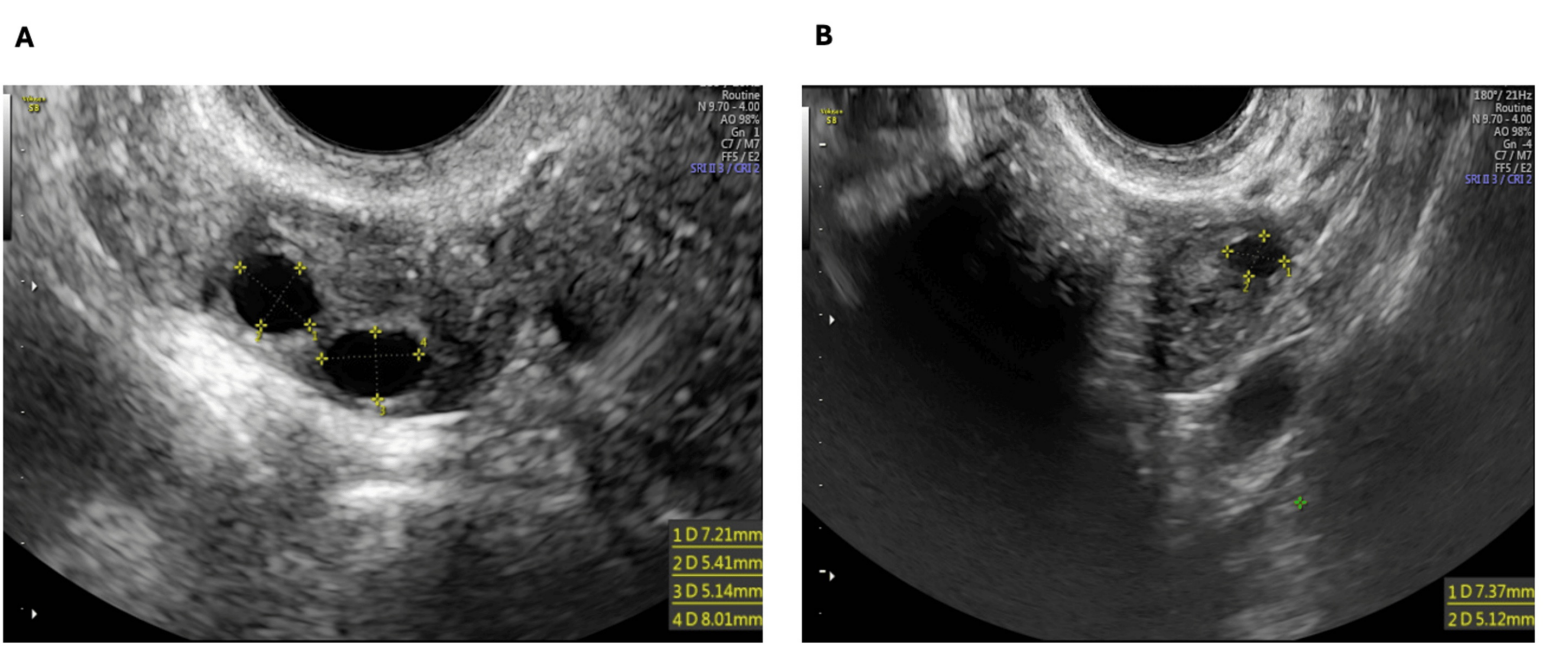
Ovarian ultrasound findings five months after the second PRP treatment. (A) Transvaginal ultrasound of the right ovary showing two antral follicles, measuring 6 mm and 7 mm in diameter, five months after the second PRP procedure. (B) Transvaginal ultrasound of the left ovary showing the presence of a single antral follicle. PRP: platelet-rich plasma

Blood tests revealed AMH at 0.03 ng/mL, FSH at 32 mIU/mL, and LH at 15 mIU/mL. On the basis of these findings, a second PRP cycle was performed on the patient. Menstruation resumed three months after the last PRP administration. Five months after the second PRP cycle, an ultrasound revealed two antral follicles in the right ovary (6 mm and 7 mm) and one antral follicle in the left ovary (Figure 2), and the hormonal values significantly improved: AMH at 0.1 ng/mL, FSH at 25 mIU/mL, and LH at 32 mIU/mL. To clearly illustrate the changes in hormonal profile throughout the PRP treatment and subsequent stimulation, the main laboratory results are summarized in Table 1.

**Table 1:** Longitudinal serum hormonal profile before, during, and after ovarian PRP treatment in a patient with primary ovarian insufficiency (POI). AMH: anti-Müllerian hormone; β-hCG: beta human chorionic gonadotropin; FSH: follicle-stimulating hormone; LH: luteinizing hormone; PRP: platelet-rich plasma

Phase/Time Point	AMH	Reference Range	FSH	Reference Range	LH	Reference Range	Comments
1 month after the first PRP	0.03 ng/mL	<1.0-9.5 ng/mL	32 mIU/mL	Follicular phase: ~1-10 mIU/mL	15 mIU/mL	Follicular phase: 1.7-15 mIU/mL	One follicle observed, right ovary
3 months after the second PRP	-	-	-	-	-	-	Menstruation resumed
5 months after the second PRP	0.1 ng/mL	<1.0-9.5 ng/mL	25 mIU/mL	Follicular phase: ~1-10 mIU/mL	32 mIU/mL	Follicular phase: 1.7-15 mIU/mL	Two follicles right, one left ovary
After embryo transfer	-	-	-	-	-	-	β-hCG: 343 mIU/mL, confirmed pregnancy

Controlled ovarian stimulation (COS)

**Figure 3:**
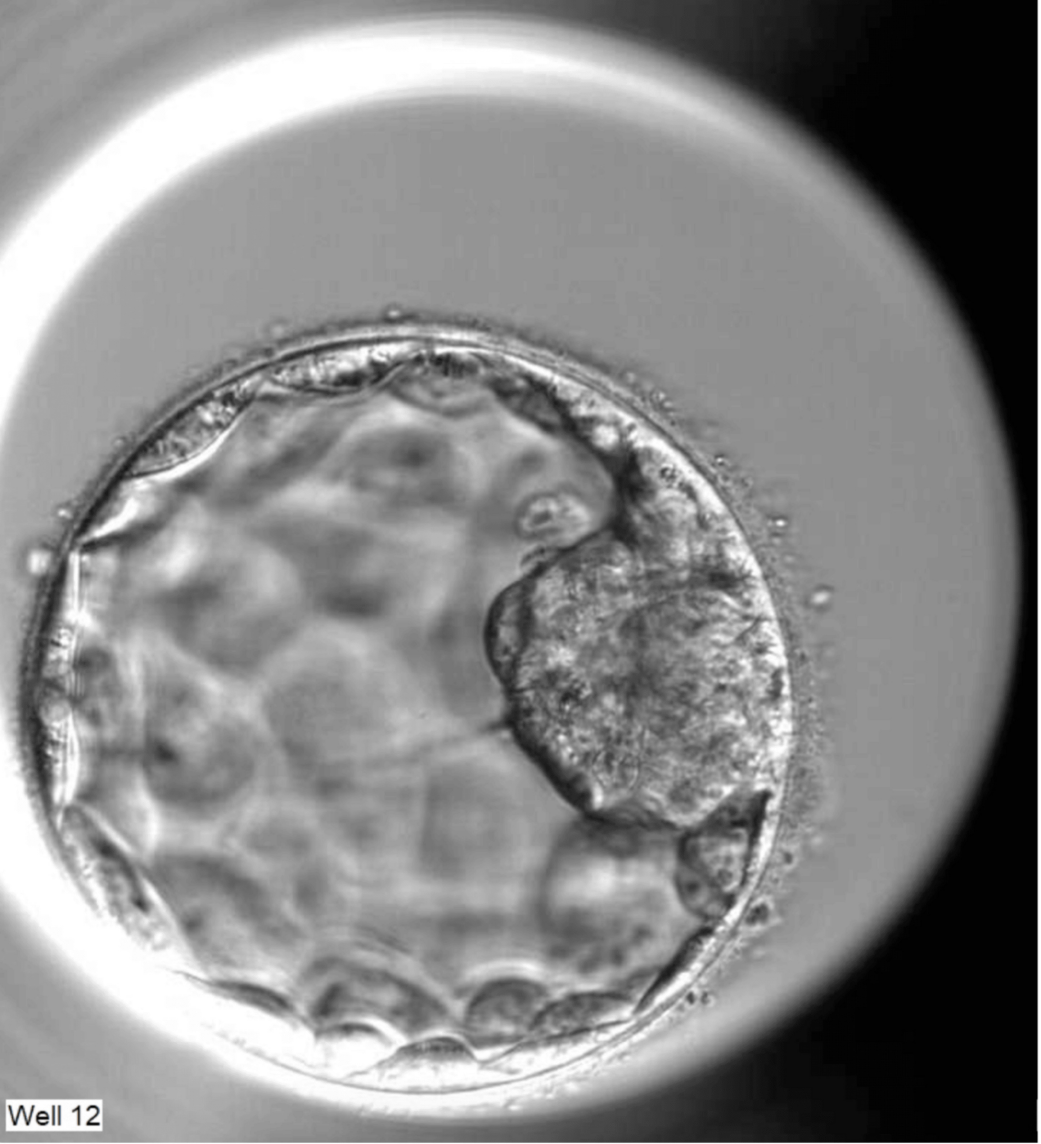
Morphology of a type 4B blastocyst on day 5 of culture.

**Figure 4:**
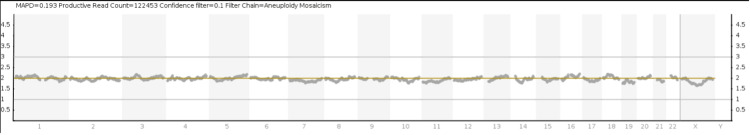
Next-generation sequencing (NGS) (Vitrolife, Gothenburg, Sweden) chromosomal analysis of a day-5 blastocyst biopsy following preimplantation genetic testing for aneuploidy (PGT-A).

Five months after the second PRP treatment, the patient underwent COS via the DuoStim protocol [8], beginning on the second day of the cycle. Individualized recombinant gonadotropin therapy, initiated with Ovaleap (Theramex Italy S.r.l., Milan, Italy) at 300 IU, was adjusted according to serial ultrasounds and serum estradiol and LH levels up to a maximum dose of 325 IU. A gonadotropin-releasing hormone (GnRH) antagonist was administered once the follicle diameter reached 14 mm. Final oocyte maturation was achieved by a GnRH analog (Fertipeptil; Ferring S.p.A., Milan, Italy) when the follicle size reached ≥17 mm, and oocyte recovery was conducted 35-36 hours later. One oocyte was retrieved and fertilized, but it degenerated by day 3 post-fertilization. Five days after the first oocyte retrieval, a second stimulation cycle was performed via the same protocol, with the final oocyte maturation triggered with 10,000 UI of human chorionic gonadotrophin (hCG) (Gonasi; IBSA Farmaceutici Italia, Lodi, Italy). Two follicles were retrieved, with one mature oocyte in metaphase II, which was fertilized correctly. A type 4B blastocyst [13] was obtained on day 5 of culture (Figure 3) and biopsied for PGT-A via next-generation sequencing (NGS) (Figure 4). PGT-A was performed at the Eurofins Genoma Group laboratory (Rome, Italy). Biopsied cells were lysed, and genomic DNA was subjected to whole-genome amplification using the SurePlex DNA Amplification System (Vitrolife, Gothenburg, Sweden), followed by library preparation with the VeriSeq NGS workflow (Vitrolife, Gothenburg, Sweden). Normalized libraries were pooled and sequenced on a MiSeq System using the MiSeq Reagent Kit v3 (Illumina, San Diego, CA, USA). Raw sequencing data were processed and analyzed with BlueFuse Multi software (Vitrolife, Gothenburg, Sweden), which generated genome-wide chromosomal copy-number profiles and aneuploidy maps used for the final clinical interpretation.

**Figure 5:**
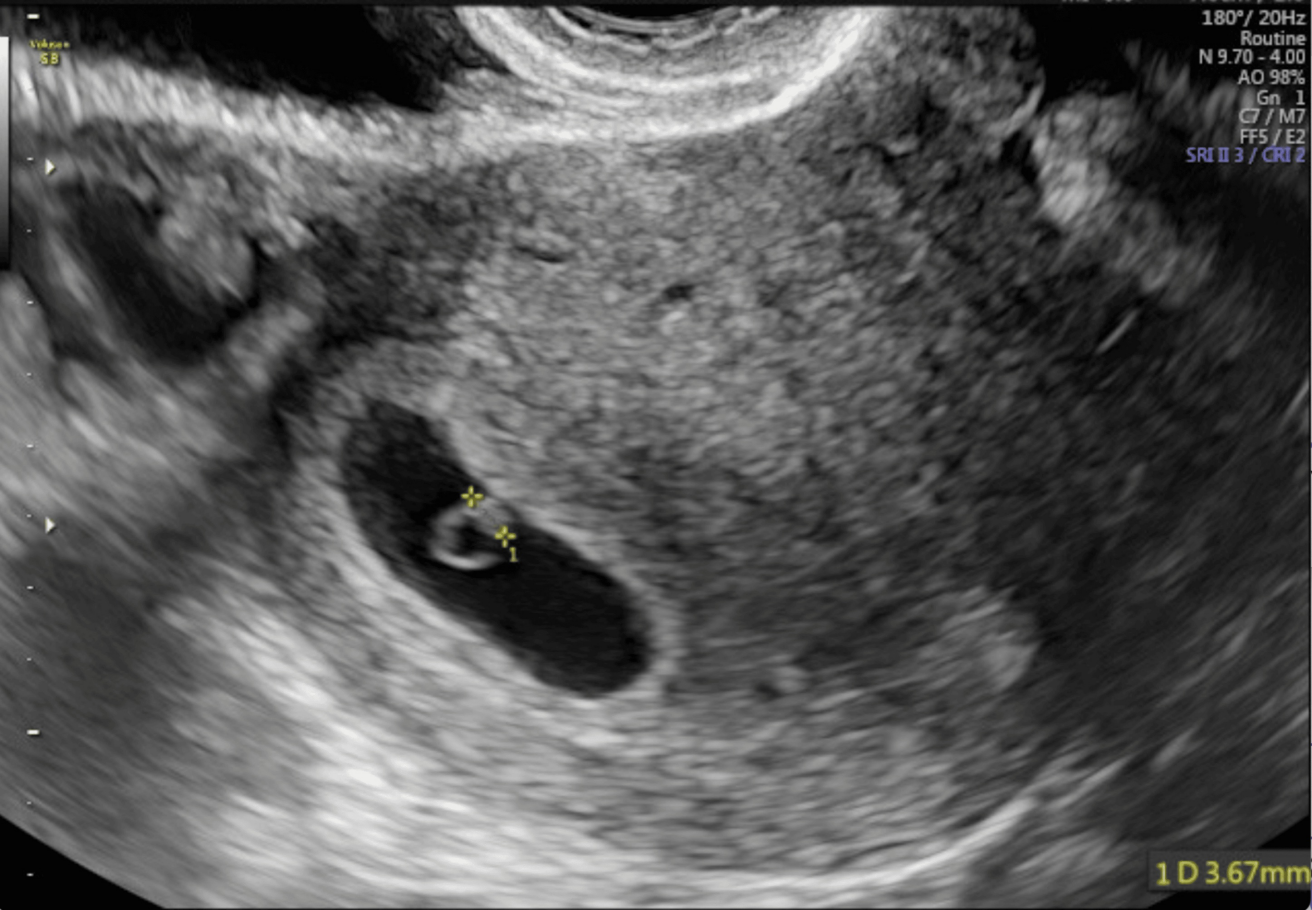
Gestational ultrasound at five weeks + one day.

The vitrified blastocyst was found to be euploid. Frozen blastocyst transfer was effectuated with a prepared endometrial protocol using hormone replacement therapy (HRT) with a GnRH agonist and oral estradiol valerate (up to 6 mg/day) from cycle day 2. The endometrial thickness reached a maximum of 8.7 mm. Luteal support was affected by vaginal progesterone (400 mg twice daily). On the sixth day of progesterone (P+5), the blastocyst was thawed and transferred under ultrasound guidance by a Wallace catheter (Smiths Medical International Ltd., Kent, United Kingdom). The serum β-human chorionic gonadotropin (β-hCG) concentration was 343 mIU/mL on day 13 after transfer, and ultrasound confirmed a singleton pregnancy (Figure 5). At 38 weeks and five days, a delivery was performed through a cesarean section because of breech presentation, and a healthy female infant weighing 3,420 g was born.

## Discussion

Intraovarian PRP has more recently emerged as a novel treatment in ART practice for patients with a DOR or POI with a low ovarian reserve [14]. One of the most significant possible advantages of PRP may be its potential to increase the euploidy rates of embryos through several biological mechanisms. From a clinical point of view, clinical data also revealed improvements in ovarian reserve parameters using autologous PRP infusion, with increased AMH and antral follicular count (AFC) and decreased FSH values [15-20]. In addition, an increase in the number of retrieved oocytes and mature oocytes after PRP treatment in a low ovarian reserve context has been detected [15,17,18]. While the above numbers are encouraging, the body of available clinical evidence remains limited.

From a molecular point of view, PRP, which is rich in growth factors, including vascular endothelial growth factor (VEGF), insulin-like growth factor-1 (IGF-1), platelet-derived growth factor (PDGF), and transforming growth factor-beta (TGF-β) [21], helps create a conducive intraovarian microenvironment for the development of the angiogenic phenomenon, rejuvenating the ovarian reservoir of stem cells and facilitating normal follicullogenesis and oocyte developmental competence [22,23].

PRP has been suggested as a favorable adjuvant in reproductive medicine because of its multiple advantages in modulating oocyte quality and embryogenesis. PRP not only is an antioxidant but also may be involved in the restoration of DNA repair and mitochondrial activity in aging or metabolically compromised areas, which may favor the genetic stability of maturing oocytes and may lead to a reduction in the risk of aneuploidy [21,24-26]. These effects are especially desirable for women with a low ovarian reserve, as PRP may increase the number and quality of resting follicle-derived oocytes, as well as the number and quality of correct spindles and chromosomes with which to work [5,27,28].

In addition, there is now early evidence that PRP can also exert a beneficial effect on the local epigenomic environment of the ovarian follicle, all of which may help preserve chromosomal dynamics during oocyte maturation and chromatid passage [27,29]. As a result, stromal oocyte connections may also be reinforced by PRP, which can provide support for nuclear and cytoplasmic maturity, a factor essential for proper chromosomal and subsequent embryonic development [30]. Together, these pathways might result in increased production of euploid embryos and thus increase live birth rates.

The timing of PRP action is also very significant. Intraovarian PRP induces the activation of growth factors in the ovarian stroma and preantral follicles, which leads to an increase in angiogenesis, the suppression of oxidative stress, and the activation of essential intraovarian pathways (e.g., the PI3K-AKT and mTOR pathways) associated with follicle pool recruitment [3]. These patients may often present with early signs of follicular activation, as indicated by the elevation of AFC or AMH (both indicators of ovarian reserve) within three to four weeks. However, full clinical benefits, particularly treatment-related improvements in oocyte and embryo quality and euploidy, may be achieved only several months after treatment [14,21]. This delayed response aligns with the average duration of folliculogenesis, which spans approximately 90-120 days from the recruitment of primordial follicles to the development of antral follicles.

## Conclusions

In conclusion, intraovarian PRP may modify the ovarian environment, oocyte quality, and embryo euploidy in patients with DOR or POI, especially in conjunction with novel ART procedures, such as DuoStim and PGT-A. Although our case contributes evidence for such a novel treatment, larger high-quality studies should be conducted to verify its effect on live birth rates and miscarriage risk and to determine the optimal protocols and patient selection in ART.
